# Sexual dimorphic response to rituximab treatment: A longitudinal observational study in a large cohort of patients with primary membranous nephropathy and persistent nephrotic syndrome

**DOI:** 10.3389/fphar.2022.958136

**Published:** 2022-09-02

**Authors:** Annalisa Perna, Barbara Ruggiero, Manuel Alfredo Podestà, Luca Perico, Silvia Orisio, Hanna Debiec, Giuseppe Remuzzi, Piero Ruggenenti

**Affiliations:** ^1^ Department of Renal Medicine, Centro di Ricerche Cliniche Aldo e Cale Daccò, Istituto di Ricerche Farmacologiche Mario Negri IRCCS, Bergamo, Italy; ^2^ Department of Health Sciences, Università Degli Studi di Milano, Milano, Italy; ^3^ Sorbonne Université and Institut National de la Santé et de la Recherche Médicale, Unité Mixte de Recherche, Paris, France; ^4^ Unit of Nephrology and Dialysis, Azienda Socio Sanitaria Territoriale Papa Giovanni XXIII, Bergamo, Italy

**Keywords:** sex, membranous nephropathy, rituximab, nephrotic syndrome, remission, anti-PLA_2_R

## Abstract

Rituximab is one of the first-line therapies for patients with membranous nephropathy (MN) at high risk of progression towards kidney failure. We investigated whether the response to Rituximab was affected by sex and anti-PLA_2_R antibody levels in 204 consecutive patients (148 males and 56 females) with biopsy-proven MN who were referred to the Nephrology Unit of the Azienda Socio Sanitaria Territoriale Papa Giovanni XXIII from March 2001 to October 2016 and managed conservatively for at least 6 months. The primary outcome was a combined endpoint of complete (proteinuria <0.3 g/24 h) or partial (proteinuria <3.0 g/24 h and >50% reduction vs. baseline) remission. Patients gave written informed consent to Rituximab treatment. The study was internally funded. No pharmaceutical company was involved. Anti-PLA_2_R antibodies were detectable in 125 patients (61.3%). At multivariable analyses, female gender (*p* = 0.0198) and lower serum creatinine levels (*p* = 0.0108) emerged as independent predictors of better outcome (*p* = 0.0198). The predictive value of proteinuria (*p* = 0.054) and anti-PLA_2_R titer (*p* = 0.0766) was borderline significant. Over a median (IQR) of 24.8 (12.0–36.0) months, 40 females (71.4%) progressed to the combined endpoint compared with 73 males (49.3%). Anti-PLA_2_R titers at baseline [127.6 (35.7-310.8) vs. 110.1 (39.9–226.7) RU/ml] and after Rituximab treatment were similar between the sexes. However, the event rate was significantly higher in females than in males [HR (95%): 2.12 (1.44–3.12), *p* = 0.0001]. Forty-five of the 62 patients (72.3%) with anti-PLA_2_R titer below the median progressed to the combined endpoint *versus* 35 of the 63 (55.6%) with higher titer [HR (95%): 1.97 (1.26–3.07), *p* < 0.0029]. The highest probability of progressing to the combined endpoint was observed in females with anti-PLA_2_R antibody titer below the median (86.7%), followed by females with anti-PLA_2_R antibody titer above the median (83.3%), males with titer below the median (68.1%), and males with titer above the median (44.4%). This trend was statistically significant (*p* = 0.0023). Similar findings were observed for complete remission (proteinuria <0.3 g/24 h) and after analysis adjustments for baseline serum creatinine. Thus, despite similar immunological features, females were more resilient to renal injury following Rituximab therapy. These findings will hopefully open new avenues to identify the molecular pathways underlying sex-related nephroprotective effects.

## Introduction

Sex impacts most organ systems in the body (Institute of Medicine Committee on Understanding the Biology of Sex and Gender Differences et al., 2001). Although investigations into sexual dimorphism in clinical research have been encouraged for years, there is still a considerable lack of knowledge about the impact of sex on human physiology and pathophysiology (NIH, 2001; Putting gender on the agenda, 2010). Sex is a strong biological variable that affects immune responses to both self and foreign antigens, with females being generally more inclined to developing most autoimmune diseases (Whitacre, 2001; Rubtsova et al., 2015; Klein and Flanagan, 2016).

However, this general rule does not seem to apply to primary membranous nephropathy (MN), a renal autoimmune disease that represents one of the most common causes of adult nephrotic syndrome (Wasserstein, 1997). MN affects females less frequently than males (Cattran, 2005), and several old observational studies have reported that disease outcomes are also generally less severe in females than in males (Hopper et al., 1981; Mallick et al., 1983; Davison et al., 1984; Tu et al., 1984; Durin et al., 1990). Data from more than 1 decade ago (Cattran et al., 2008) revealed that the rate of renal function loss was slower, and kidney survival was longer in younger females with MN than in males. Blood pressure and proteinuria at disease presentation were also lower in females than in males. However, the positive effect of the female sex persisted in multivariable analyses adjusting for these covariates. *Post hoc* analyses of the Ramipril and Efficacy trial in nephropathy (Ruggenenti et al., 2000) also showed that the Angiotensin Converting Enzyme (ACE) inhibitor therapy was more nephroprotective in females than in males largely because Ramipril reduced proteinuria and the rate of GFR decline in females independently of their ACE polymorphism. In contrast, the treatment effect in males was restricted only to those with the DD genotype. This is consistent with evidence indicating that, independent of treatment, males are more susceptible to developing chronic kidney disease and progressing to end-stage renal disease than females (Neugarten et al., 2000; Iliescu and Reckelhoff, 2008; USRDS, 2007, Annual Data Report). Thus, understanding how sex-based differences in immunity may affect the onset and response to therapy of primary MN is of crucial interest to get a better insight into the pathophysiology of the disease and provide the most appropriate clinical management for both sexes.

A major contribution to understanding the pathophysiology of MN derived from the landmark work by Debiec et al. (2002) who first identified a target antigen in the baby of a woman with a neutral endopeptidase (NEP) deficiency. The finding that anti-NEP alloantibodies produced by the mother crossed the placenta and bound to NEP expressed on fetal podocytes confirmed the role of autoantibodies in the pathogenesis of primary MN in humans. Then, Beck et al. (2009) reported that the M-type phospholipase A2 receptor (PLA_2_R) is the major antigen in primary MN and revealed the presence of anti-PLA_2_R autoantibodies in most MN patients. This milestone discovery has been widely confirmed in subsequent studies showing that 53%–80% of patients with primary MN exhibited circulating and glomerular anti-PLA_2_R autoantibodies (Hofstra et al., 2011; Qin et al., 2011; Oh et al., 2013; Akiyama et al., 2015; Dong et al., 2016; Hihara et al., 2016) reviewed by Ruggenenti et al. (2017). The pathogenic role of anti-PLA_2_R autoantibodies was confirmed by finding that the antibody titer strongly correlated with disease activity in most study populations (Kim et al., 2015; Kumar et al., 2015; Radice et al., 2016; Ramachandran et al., 2016). Additionally, anti-PLA_2_R antibody titer also predicted the response to therapies such as Rituximab (Beck et al., 2011; Cravedi et al., 2011a; Ruggenenti et al., 2015), a monoclonal antibody against the B-cell surface antigen CD20. Indeed, Rituximab is a valuable option for the treatment of primary MN (Remuzzi et al., 2002; Cravedi et al., 2007; Ruggenenti et al., 2008; El-Zoghby et al., 2009; Cravedi et al., 2010; Ruggenenti et al., 2012; Cravedi et al., 2014) because direct targeting of B cells could be exploited to inhibit the production of anti-PLA_2_R nephritogenic autoantibodies. Consistently, after the first successful report (Remuzzi et al., 2002), cohort studies showed that Rituximab could induce nephrotic syndrome (NS) remission in a significant fraction of MN patients (Ruggenenti et al., 2012). This effect was predicted by Rituximab-induced depletion of circulating anti-PLA_2_R antibodies (Ruggenenti et al., 2015)**.** Subsequently, randomized controlled trials (RCTs) confirmed the superiority of Rituximab in inducing and maintaining NS remission compared to either supportive therapy alone (Dahan et al., 2017) or cyclosporine (Fervenza et al., 2019)**.** In recent years, in a randomized pilot trial of 74 patients with new incident primary MN (Scolari et al., 2021), Rituximab (two 1 g doses, 2 weeks apart) achieved an 85% of a 3-year rate of complete or partial remissions compared to the 73% rate achieved by cyclophosphamide and steroid combination therapy (HR [95% CI] 2.12 [0.45–9.96]). Notably, three cases of leukopenia and three cases of pneumonia were reported in the combined treatment group compared to no cases in the Rituximab group. On the basis of the findings of the aforementioned trials, Rituximab has progressively become one of the first-line therapies for primary MN (Ruggenenti et al., 2017; Sabiu and Podestà, 2021)**,** even if the cyclic cyclophosphamide and corticosteroid combined treatment protocol continues to be used in several Nephrology Units.

Thus, in this study, we sought to investigate whether and to what extent the response to Rituximab could be affected by sex and anti-PLA_2_R antibody levels in a large and well-defined cohort of consecutive patients with primary MN and persistent NS.

## Methods

### Study population and treatment

All consecutive patients with biopsy-proven MN referred to the Nephrology Unit of the Azienda Socio Sanitaria Territoriale (ASST) Ospedale Papa Giovanni XXIII from March 2001 to October 2016, who had proteinuria persistently >3.5 g/24 h despite at least 6-month supportive therapy including ACE inhibitors and/or angiotensin receptor blockers (ARBs) but no steroid or any immunosuppressive medication, creatinine clearance >20 ml/min per 1.73 m^2^, and no evidence of secondary MN or circulating hepatitis B surface antigens, were treated with Rituximab. As previously described (Ruggenenti et al., 2015), up to October 2005, patients received four 375 mg/m^2^ weekly doses of Rituximab. After that, they received a first Rituximab infusion of 375 mg/m^2^ followed by a second infusion only when circulating B cells >5 mm^3^ were detected 1 week after completion of the first Rituximab administration (“B-cell-driven” protocol). This approach was dictated by evidence that the two treatment regimens were associated with similar outcomes (Cravedi et al., 2007)**.** Patients gave written informed consent to Rituximab treatment and laboratory analyses according to the Declaration of Helsinki guidelines. Rituximab was supplied by the pharmacy of the ASST. The study was internally funded, and no pharmaceutical company was involved.

### Monitoring

Before Rituximab administration, proteinuria was measured in three consecutive 24 h urine collections, and the average value was recorded. Creatinine excretion was measured on the day of the last collection. A blood sample was collected for hematochemistry and blood cell counts. Clinical and laboratory parameters evaluated at baseline were then evaluated at months 1, 2, 3, 6, 9, and 12 after Rituximab administration and at least every 6 months thereafter, for up to 5 years. The glomerular filtration rate (GFR) was directly measured by the Iohexol plasma clearance technique (Gaspari et al., 1995) immediately before Rituximab administration and every 6 months thereafter.

### Anti-PLA_2_R autoantibody evaluation

Circulating anti-PLA_2_R1 total IgG antibodies were measured at baseline (before Rituximab administration) and at subsequent time points during the follow-up. After sampling, all sera were immediately aliquoted, frozen, and stored at –20°C. After thawing, all serum samples were tested for the presence of anti-PLA_2_R total IgG antibodies using a validated quantitative ELISA test kit (EUROIMMUN, Lübeck, Germany) (Dahnrich et al., 2013). In brief, sera diluted to 1:100 were incubated with PLA_2_R coated microplates and detected with anti-human IgG horseradish peroxidase conjugate. The final concentrations for each sample were calculated from the calibration curve extinction values plotted against the concentration for each calibrator. Anti-PLA_2_R antibody values ≥1 RU/ml were considered to identify patients with detectable anti-PLA_2_R antibodies. These values were included in analyses considering anti-PLA_2_R titer as a continuous variable. Anti-PLA_2_R antibody values = 0 RU/ml were considered to identify patients with undetectable anti-PLA_2_R antibodies. The coefficients of variation (CV) were assessed using three selected serum samples covering the measuring range. The intra-assay and interassay CVs were determined on 20 measurements for each serum in one set or on a threefold replica in ten sets, respectively. In our laboratory, the calculated intra- and interassay CVs are <4% and <9%, respectively. Up to five freeze/thaw cycles were found not to affect anti-PLA_2_R binding by ELISA**.**

### Outcomes

The primary outcome was a combined endpoint of complete (proteinuria <0.3 g/24 h) or partial (proteinuria <3.0 g/24 h and >50% reduction vs. baseline) remission of nephrotic proteinuria. Complete remission of nephrotic proteinuria (proteinuria <0.3 g/24 h), considered a single endpoint, was the primary outcome of sensitivity analyses. Secondary outcomes included a relapse of proteinuria, defined as proteinuria increase to >3.5 g/24 h in subjects with previous complete or partial remission. Other outcomes included post-Rituximab changes in continuous variables such as anti-PLA_2_R titer.

### Statistical analyses

All patients with at least 6 months of follow-up were considered for the analyses. Baseline parameters were shown as frequency counts or percentages and as means and SDs or medians and IQRs according to normal or skewed data distribution, respectively. Groups were compared by one-way analysis of variance (ANOVA), the Kruskal–Wallis test, Pearson’s chi-square test, Fisher’s exact test, non-parametric test for trend, and analysis of covariance (ANCOVA) as appropriate.

Predictors of progression to the composite endpoint or complete remission were modeled by univariable and multivariable Cox regression analyses. The univariable model included the baseline variables listed in Table 1. Consistent with the purposes of the study, the multivariable Cox model included “*a priori*” established clinical risk factors, that is, severity and duration of proteinuria, along with anti-PLA_2_R1 titer, and variables that were significantly associated with the outcome in the univariable model. When different variables significantly associated with the outcome appeared to be highly correlated, the one considered the most relevant from a clinical or pathophysiological perspective (as for proteinuria vs. serum creatinine, albumin, or lipids) was selected for multivariable analyses. The maximum number of independent variables to be included in the multivariable Cox models was assessed by considering the number of outcome events per independent variable according to Peduzzi et al. (Peduzzi et al., 1995). To corroborate the results of the multivariable model, we applied the “least absolute shrinkage and selection operator” (LASSO) statistical approach for variable selection, a learning technique that very efficiently sorts relevant from irrelevant variables (Tibshirani, 1997). LASSO regression is useful in the presence of some collinearity when the assumption of independence is not fully met. We selected the four variables of interest (gender, serum creatinine, proteinuria, and anti-PLA_2_R1 titer) by using the package “ncvreg” under R (v 3.5.1); the LASSO method identified the most significantly predictive variables. The Kaplan–Meier method was used to plot the probability of progressing to the primary endpoint complete remission, the composite endpoint of complete and partial remission, or relapse after initial remission. Survival time was determined from the beginning of the first treatment until the event of interest. Patients not achieving remission were considered as censored at the time of the last visit with a non-missing value of proteinuria.

**TABLE 1:** Baseline characteristics and follow-up duration of patients in the study group as a whole (overall) and considered separately according to sex and to anti-PLA_2_R below or above the median.

Characteristic	Overall	Sex		Anti-PLA_2_R titer	
		Males	Females	Below median	Above median
	(*n* = 204)	(*n* = 148)	(*n* = 56)	(*n* = 62)	(*n* = 63)
Age (years)	52.6 ± 15.5	52.6 ± 14.7	52.7 ± 17.6	54.3 ± 15.0	55.7 ± 13.1
Male gender (*n* %)	148 (72.5%)	148 (100%)	0	47 (75.8%)	45 (71.4 %)
*Clinical parameters*					
BMI (kg/m^2^)	26.9 ± 4.6	26.8 ± 3.8	27.4 ± 6.4	26.5 ± 4.1	27.2 ± 5.0
Systolic blood pressure (mmHg)	143.2 ± 17.5	135.2 ± 17.1	131.6 ± 18.4	135.0 ± 17.8	136.8 ± 16.9
Diastolic blood pressure (mmHg)	80.8 ± 9.8	82.4 ± 9.8	76.6 ± 8.6°°°	80.0 ± 9.1	81.5 ± 9.2
Reported duration of proteinuria (median, mo)	24.9 (11.0–64.6)	24.7 (10.9–68.6)	25.4 (11.5–47.0)	25.3 (10.6–83.0)	18.4 (11.5–37.9)
(mean, mo)	51.6 ± 67.7	52.6 ± 69.0	48.6 ± 64.7	61.9 ± 81.6	32.9 ± 40.4
*Laboratory parameters*					
Serum creatinine (mg/dl)	1.4 ± 0.7	1.5 ± 0.8	1.1 ± 0.4°	1.3 ± 0.5	1.5 ± 0.7
Serum albumin (g/dl)	2.4 ± 0.7	2.4 ± 0.8	2.4 ± 0.6	2.2 ± 0.6	2.0 ± 0.5
Serum proteins (g/dl)	4.8 ± 0.9	4.8 ± 1.0	4.8 ± 0.7	4.6 ± 0.7	4.4 ± 0.7
Total cholesterol (mg/dl)	266.0 ± 82.6	262.9 ± 82.8	274.2 ± 82.1	274.7 ± 75.5	277.2 ± 88.4
HDL cholesterol (mg/dl)	56.3 ± 19.1	51.8 ± 15.2	68.2 ± 23.1°°°	56.0 ± 17.0	54.5 ± 18.4
Triglycerides (mg/dl)	179.6 ± 104.7	191.9 ± 113.1	147.3 ± 69.5°°	166.3 ± 90.9	199.6 ± 124.7
Proteinuria (g/24 h)	8.3 (4.9–12.0)	9.2 (5.9–13.0)	5.8 (3.8–8.4)°°°	8.6 (5.6–11.8)	11.0 (7.7–13.6)*
Iohexol-measured GFR (ml/min/1.73 m^2^)	62.3 ± 25.8	63.6 ± 26.2	59.2 ± 24.9°	60.2 ± 23.3	55.5 ± 19.0
Anti-PLA_2_R1					
Titer (RU/ml)	217.3 ± 489.8	159.1 ± 178.1	387.4 ± 908.5	69.3 ± 35.9	445.7 ± 691.5 ***
Detectable/undetectable/unavailable	125/24/55	92/19/37	33/5/18	-	-
Follow-up (mo)	24.8 (12.0–36.0)	24.0 (12.3–36.0)	17.4 (10.1–36.0)	22.0 (12.0–36.0)	24.1 (15.0–37.6)

Variables are expressed as mean ± SD, or as median and IQR, or both. Categorical variables are expressed as a number (percentage).

°*p* < 0.05, °°*p* < 0.01, °°°*p* < 0.001 *versus* males; *p < 0.05, ***p < 0.001 V S, below anti-PLA2R median (unpaired *t*-test or Wilcoxon rank-sum test, as appropriate).

## Results

**FIGURE 1:**
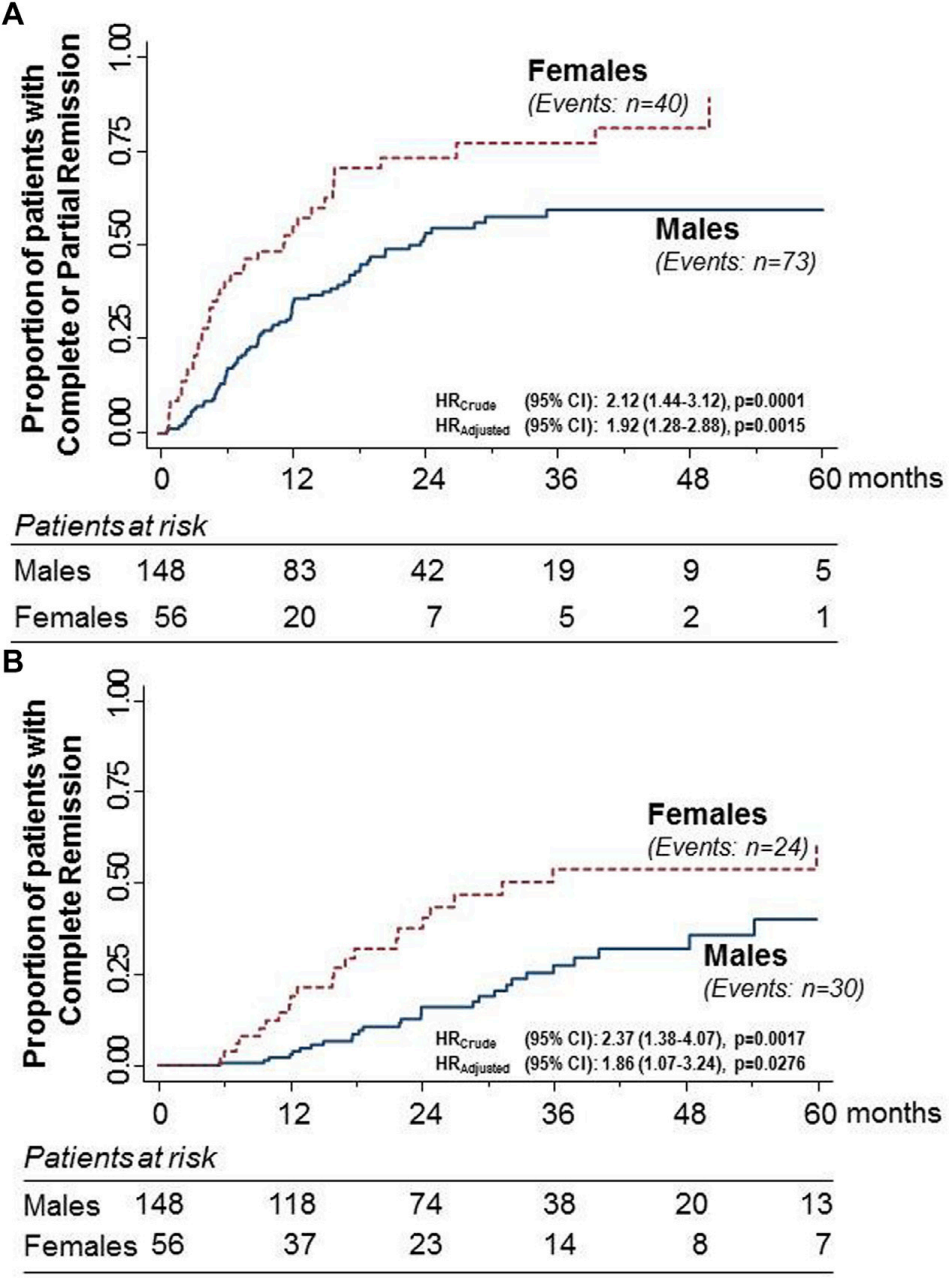
Kaplan–Meier curves for the proportion of participants with primary MN who progressed to the combined endpoint of complete or partial remission **(A)** or to complete remission considered as a single endpoint **(B)** in the subgroups of males and females considered separately. The rate of progression to both endpoints was significantly higher in females than in males. The difference was significant even after adjusting for baseline serum creatinine levels.

Overall, 204 patients had biopsy-proven primary MN and were treated with Rituximab monotherapy (Figure 1). Per protocol, all of them had persistent proteinuria >3.5 g/24 h for at least 6 months without previous spontaneous remissions, and none of them received steroids or any other immunosuppressant for at least 6 months before Rituximab administration. Rituximab was the first-line therapy in 110 patients (53.9%). Of the 204 patients, 148 were males (72.5%) and 56 were females (27.5%). Sera for the determination of anti-PLA_2_R antibody levels were available for 149 patients (73.0%). Anti-PLA_2_R antibodies were detectable in 125 patients: 92 of the 111 tested males (82.9%) and 33 of the 38 tested females (86.8%). All study patients were considered in the analyses.

### Baseline characteristics according to sex and anti-PLA_2_R antibody titer

Selection criteria are patients with heavy proteinuria, hypoalbuminemia/hypoproteinemia, and dyslipidemia at inclusion. Proteinuria was significantly higher (almost double) in males than in females (*p* < 0.001), whereas the reported duration of proteinuria was similar between the two groups. Dyslipidemia was more severe in males with higher diastolic blood pressure values than females (Table 1). Conversely, hypoalbuminemia and hypoproteinemia were similarly severe in both sexes. On average, patients had moderate renal insufficiency with measured GFR significantly lower in females (Table 1). Thus, the significantly higher serum creatinine levels observed in males despite slightly higher GFR most likely reflected the higher muscular mass of males compared to females.

Baseline characteristics of patients with anti-PLA_2_R titer below or above the median were similar, with the sole exception of proteinuria, which was higher in patients with anti-PLA_2_R titer above the median compared with those with a lower antibody level (Table 1).

### Outcome predictors

The 204 patients were followed up for a median (IQR) of 24.8 (12.0–36.0) months after Rituximab treatment. Overall, 113 patients (55.4%) progressed to the combined endpoint of complete or partial remission over a median (IQR) of 7.8 (4.1–14.8) months, whereas 54 patients (26.5%) achieved complete remission, considered as a single endpoint over 21.7 (12.2–31.7) months.

Covariates that at univariable and multivariable analyses were associated with progression to complete or partial remission, as shown in Table 2. At univariable analyses, the female gender emerged as a strong predictor of increased probability of progressing to the combined endpoint. Other predictors of better outcomes were lower diastolic blood pressure and less severe proteinuria, as well as lower serum triglyceride and creatinine levels. The predictive value of anti-PLA_2_R was borderline significant. In multivariable analyses, the female gender again emerged as a strong predictor of better outcomes, in addition to lower serum creatinine levels. The predictive value of proteinuria and anti-PLA_2_R levels was borderline significant (Table 2). The LASSO method identified gender and serum creatinine as the most significant predictive variables. Univariable and multivariable analyses of baseline predictors of complete remission considered as a single endpoint were poorly informative because of the too small number of events.

**TABLE 2:** Univariable and multivariable Cox analysis for evaluating the association between considered covariates at inclusion and complete or partial remission of the nephrotic syndrome.

Characteristics	Univariable		Multivariable	
	Hazard ratio (95% CI)	*p*-value	Hazard ratio (95% CI)	*p*-value
Demography and clinical parameters				
** **Age (years)	1.003 (0.991–1.016	0.6050	—	—
** **Sex (M/F)	2.120 (1.439–3.124)	0.0001	1.823 (1.100–-3.021)	0.0198
** **BMI (kg/m2)	1.016 (0.973–1.061)	0.4654	—	—
** **Systolic BP (mmHg)	0.993 (0.982–1.004)	0.2321	—	—
** **Diastolic BP (mmHg)	0.980 (0.961–0.999)	0.0396	1.001 (0.977-–1.027)	0.9140
** **Reported duration of proteinuria (mo)	0.998 (0.996–1.001)	0.3002	—	—
Laboratory parameters				
** **Serum creatinine (mg/dL)	0.604 (0.432–0.844)	0.0031	0.570 (0.370–0.878)	0.0108
** **Serum albumin (g/dL)	1.092 (0.842–1.417)	0.5066	—	—
** **Serum proteins (g/dL)	1.059 (0.855–1.312)	0.6010	—	—
** **Total cholesterol (mg/dL)	0.998 (0.995 – 1.000)	0.0680	—	—
** **HDL cholesterol (mg/dL)	1.006 (0.996–1.017)	0.2469	—	—
** **Triglycerides (mg/dL)	0.995 (0.993–0.998)	0.0002	0.996 (0.994–0.999)	0.159
** **Proteinuria (g/24 h)	0.952 (0.920–0.985)	0.0043	0.951 (0.904–1.001)	0.054
** **Iohexol measured GFR (ml/min/1.73 m^2^)	1.001 (0.994–1.009)	0.7346	—	—
Anti-PLA_2_R1 parameters				
** **Anti-PLA_2_R1 titer (RU/ml)	0.999 (0.998–1.00)	0.0912	0.999 (0.999–1.00)	0.0766

### Outcomes according to gender and anti-PLA_2_R titer

**FIGURE 2:**
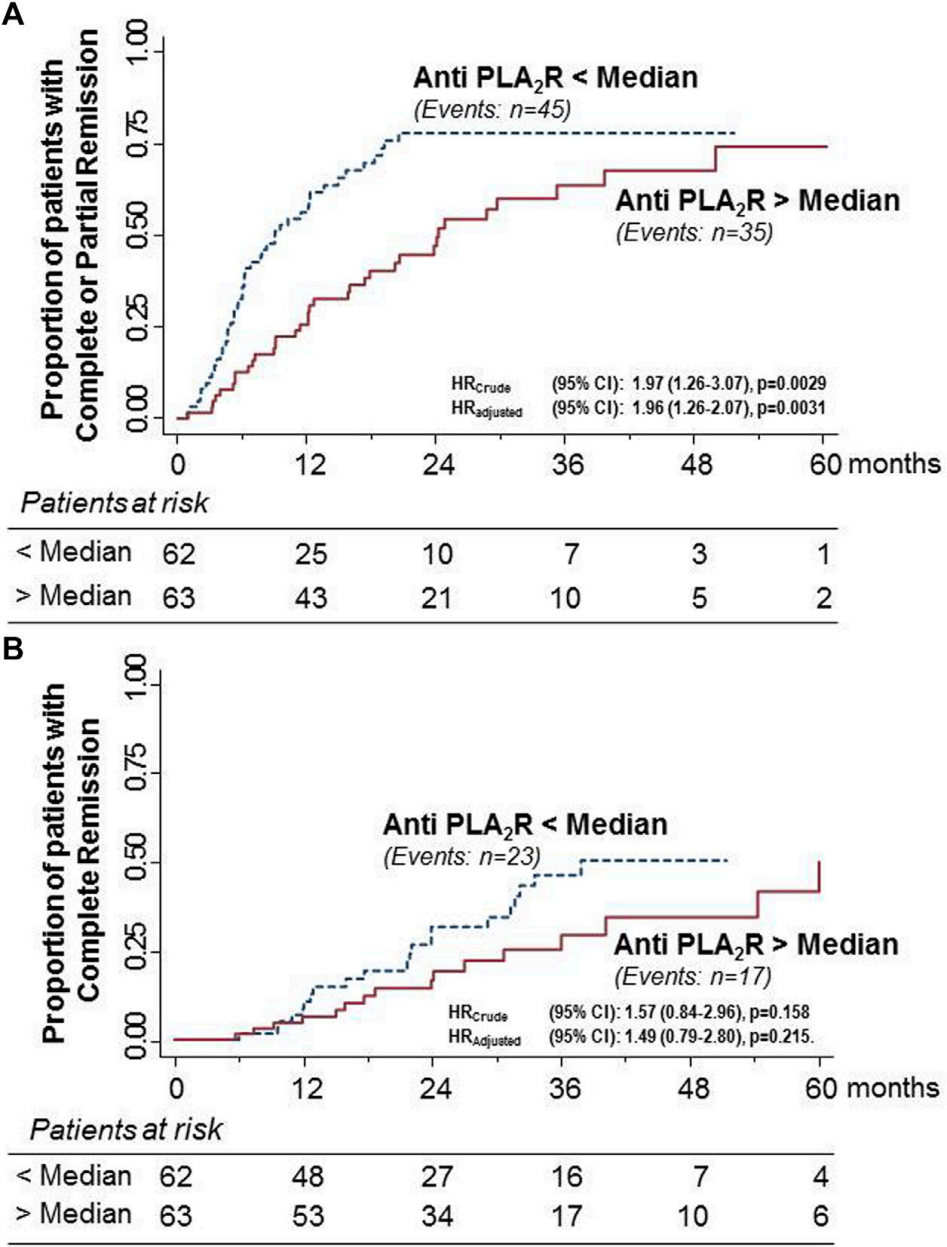
Kaplan–Meier curves for the proportion of participants with primary MN who progressed to the combined endpoint of complete or partial remission **(A)** or to complete remission considered as a single endpoint **(B)** in the subgroups of patients with anti-PLA_2_R antibody titer above or below the median considered separately. The rate of progression to the combined endpoint was significantly higher in females than in males. The difference was significant even after adjusting for baseline serum creatinine levels. Similar but non-significant trends were observed for complete remission, considered as a single endpoint, even after adjustment for baseline serum creatinine levels.

During the study period, 40 of the 56 females (71.4%) progressed to the combined endpoint compared to 73 of the 148 males (49.3%). The event rate was significantly higher in females than in males [HR (95%): 2.12 (1.44–3.12), *p* = 0.0001] (Figure 1A). The difference between the sexes was significant even after adjusting for baseline serum creatinine [HR (95%): 1.82 (1.28–2.88), *p* = 0.0015]. During the same observation period, 24 females (42.9%) progressed to complete remission, considered a single endpoint compared with 30 males (20.3%). Again, the event rate was significantly higher in females than in males [HR (95%): 2.37 (1.38–4.07), *p* = 0.0017] (Figure 2B). The difference between the sexes was significant even after adjusting for baseline serum creatinine [HR (95%): 1.86 (1.07–3.24), *p* = 0.0276].

**FIGURE 3:**
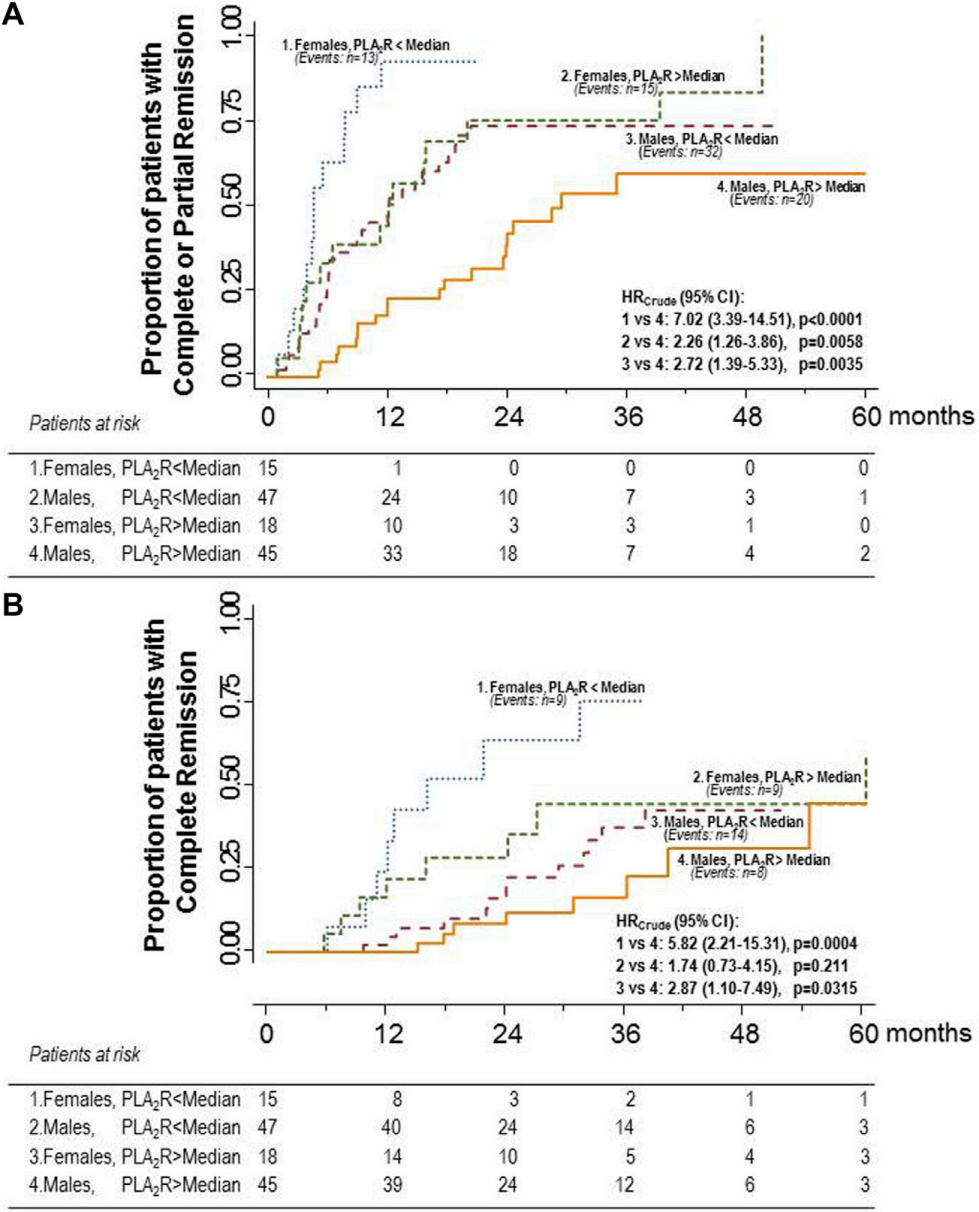
Kaplan–Meier curves for the proportion of participants with primary MN who progressed to the combined endpoint of complete or partial remission **(A)** or to complete remission considered as a single endpoint **(B)** in the four subgroups of male or female patients with anti-PLA_2_R antibody titer above or below the median considered separately. The highest probability of progressing to the combined endpoint was observed in females with anti-PLA_2_R antibody titer below the median. A slightly lower probability of progressing to the combined endpoint was observed in females with anti-PLA_2_R antibody titer above the median, whereas the probability crumbled in males with antibody titer below the median. The lowest probability was observed in males with antibody titer above the median. Differences between considered subgroups *versus* the subgroup of males with antibody titer below the median (reference group) were significant. A similar trend was observed for complete remission, considered as a single endpoint.

During the study period, 45 of the 62 patients (72.3%) with anti-PLA_2_R titer below the median progressed to the combined endpoint compared with 35 of the 63 patients (55.6%) with a titer above the median. The event rate was significantly higher in patients with lower anti-PLA_2_R titer than in those with higher levels [HR (95%): 1.97 (1.26–3.07), *p* < 0.0029] (Figure 3A). The difference between the two groups was significant even after adjusting for baseline serum creatinine [HR (95%): 1.96 (1.26–2.07), *p* = 0.0031]. During the same observation period, 23 patients (37.1%) with anti-PLA_2_R titer below the median progressed to the single endpoint of complete remission compared to 17 (27.0%) patients with higher antibody titer. The difference between the two groups failed to achieve statistical significance, even after adjustment for baseline serum creatinine levels (Figure 3B).

When patients were stratified by sex and anti-PLA_2_R titer, the highest probability of progressing to the combined endpoint was observed in females with anti-PLA_2_R antibody titer below the median (86.7%). A slightly lower probability of progressing to the combined endpoint was observed in females with anti-PLA_2_R antibody titer above the median (83.3%), whereas the probability crumbled in males with antibody titer below the median (68.1%). The lowest probability was observed in males with antibody levels above the median (44.4%). This trend was statistically significant (*p* = 0.0023). The hazard ratios of the probabilities of progressing to the composite endpoint of different groups compared to the group with the worst outcome (reference group) are shown in Figure 3A. The probability of progressing to complete remission considered as a single endpoint followed the same trend observed for the primary, combined endpoint (Figure 3B). The same trends were observed when the hazard ratios were adjusted by serum creatinine levels (data not shown). Notably, the two women with anti-PLA_2_R antibody titer above the median who failed to progress to complete or partial remission after Rituximab therapy were 59 and 80 years old, and the three women with antibody titer below the median who also failed to achieve the combined endpoint were 60, 64, and 73 years old, respectively.

### Longitudinal analyses of anti-PLA_2_R titer considered a continuous variable

**FIGURE 4:**
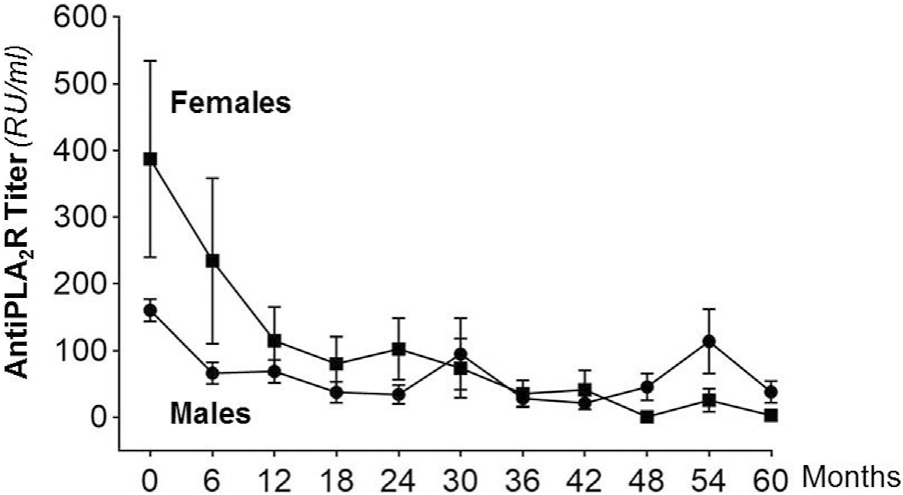
Anti-PLA_2_R antibody levels (mean ± SEM) at baseline (month 0) and at different time points after Rituximab administration in males and females considered separately. Between-group changes in antibody titer at different time points *versus* baseline never differed significantly.

The titer of circulating anti-PLA_2_R antibodies promptly decreased after Rituximab administration. In both sexes, it achieved the nadir at 6–12 months after treatment and stabilized thereafter. Changes in antibody levels *versus* baseline at each time point considered never differed between males and females (Figure 4).

### Relapses

**FIGURE 5:**
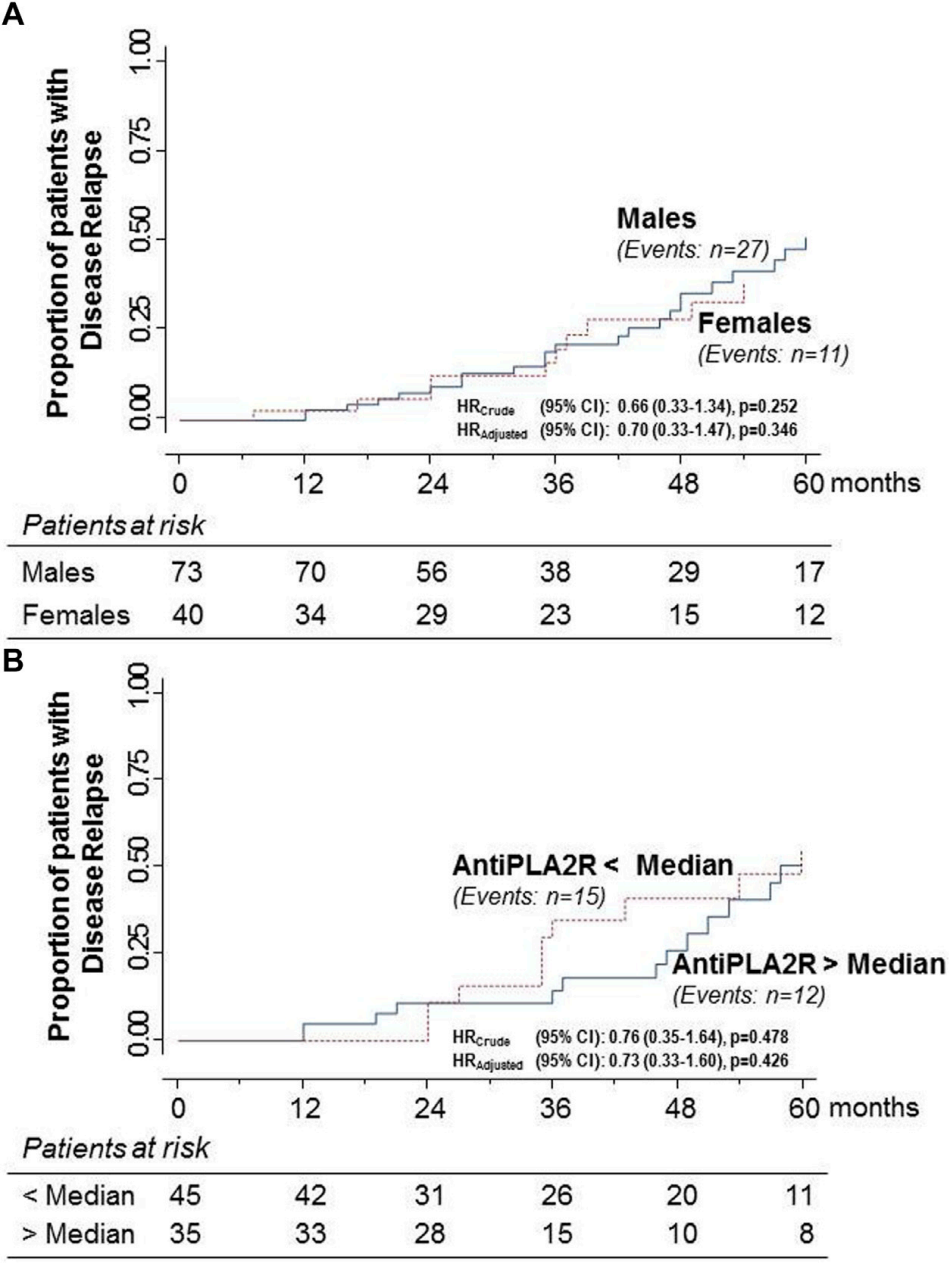
Kaplan–Meier curves for the proportion of participants with primary MN who had a relapse of the NS after progression to the combined endpoint of complete or partial remission in males and females **(A)** and in patients with anti-PLA_2_R antibody titer above or below the median **(B)** considered separately. Relapse rates never differed significantly between considered groups.

Overall, 38 of the 113 patients (33.6%) who had achieved the combined endpoint had an NS relapse over a median (IQR) of 38.3 (24.1–53.1) months after Rituximab administration. Twenty-seven of the relapsers were males (37%) and 11 females (27.5%). The proportion of relapsers was slightly higher in males than in females, but the hazard ratio for the event rate between sexes was non-significant, even after adjustment for baseline serum creatinine (Figure 5A). There were 15 relapsers among the 45 patients with anti-PLA_2_R titer below the median (33.3%) and 12 among the 35 patients with anti-PLA_2_R titer above the median (34.3%). Again, the hazard ratio for the event rate between the two groups was non-significant, even after adjustment for baseline serum creatinine (Figure 5B).

## Discussion

In this study, we sought to investigate the integrated role of sex and anti-PLA_2_R antibody titer as predictors of the response to anti-CD20 cytolytic therapy with Rituximab in more than two hundred patients with MN and persistent NS. Data showed that sex and antibody levels are important predictors of disease outcome upon Rituximab exposure. The large majority of the 56 females (71.4%) achieved the combined endpoint of complete or partial remission, and only 16 of the 56 females (28.6%) failed Rituximab therapy. Conversely, only 73 of the 148 males progressed to the same combined endpoint, whereas more than 50% failed to achieve complete or partial remission. The robustness of the findings was confirmed by the results of sensitivity analyses showing that almost half of females achieved the single endpoint of complete remission, a proportion that exceeded by more than two folds the proportion of males achieving the same endpoint. The predictive value of anti-PLA_2_R titer was confirmed by finding that 72.6% of patients with anti-PLA_2_R titer below the median achieved complete or partial remission compared with only 55.6% of those with an antibody titer exceeding the median. The hypothesis of an integrated predictive role of sex and anti-PLA_2_R antibody titer was corroborated by results of sensitivity analyses showing a similar trend when complete remission was considered as a single endpoint. However, this trend failed to achieve statistical significance most likely because of the relatively small number of events. Further analyses confirmed the integrated role of both risk factors. Indeed, when patients were categorized according to either sex and antibody titer, females with lower anti-PLA_2_R antibody titer were found to be the patients with the highest probability of achieving complete or partial remission after Rituximab treatment, a probability that approximated 90%. The probability was lower for females with higher antibody titer and males with lower antibody titer. The lowest probability that hardly exceeded 50% was observed in males with higher antibody titer. A similar trend was observed in sensitivity analyses considering complete remission as a single endpoint. Sex and antibody titer did not significantly affect the risk of relapse of the NS after the initial response to Rituximab, although the proportion of relapsers appeared to be slightly higher in males than in females. On the contrary, the very low rate of relapses provided additional evidence of the remarkable efficacy profile of Rituximab in the treatment of primary MN (Ruggenenti et al., 2008; Ruggenenti et al., 2012)**.**

Why do sex and antibody titer have such a strong impact on disease outcome upon exposure to Rituximab? The prognostic value of anti-PLA_2_R levels has already been reported and discussed elsewhere (Cravedi et al., 2011b; Ruggenenti et al., 2015)**.** Conversely, data on the prognostic impact of sex in this context are fully novel. Despite between-sexes similar anti-PLA_2_R levels at inclusion, female patients had a less aggressive disease phenotype, as indicated by lower levels of proteinuria along with significantly less severe dyslipidemia. However, less disease severity at inclusion only partially explained the better outcomes for females because multivariable analyses showed that sex retained a significant predictive value for the progression to the combined endpoint of complete or partial remission even after adjusting for the potential confounders, including anti-PLA_2_R titer. Similar results were obtained using the LASSO statistical approach. Thus, sex-related factors appear to play an independent and specific role in the progression of MN after Rituximab therapy, a role that is unlikely explained by sex-related differences in the humoral response of the immune system to Rituximab. Indeed, changes in anti-PLA_2_R titer upon exposure to Rituximab never differed appreciably between males and females. This finding, as well as evidence that baseline anti-PLA_2_R antibody titer was similar in males and females, appears to suggest that less severe disease phenotype at inclusion and better outcomes in response to Rituximab could indicate that females with primary MN might be able to better manage and resolve similar immunological challenges compared with males. The hypothesis that, independent of anti-PLA_2_R antibody titer, sex hormones have a major impact on the larger benefit of Rituximab therapy in females than in males was corroborated by finding that the two women with a titer below the median and the three women with a titer above the median who failed to achieve complete or partial remission after Rituximab administration were all in the postmenopausal age.

Several observations in both rodents and humans provide additional clues to this issue. Indeed, the same experimental models of chronic kidney disease are associated with slower progression to end-stage renal disease in the female than in male animals (Yanes et al., 2008). Strikingly, specific strains of rats develop proteinuric, progressive nephropathies in male animals only, whereas female animals are fully protected from renal disease (Remuzzi et al., 1988)**.** In humans, the progression of the same immune-mediated kidney diseases is less aggressive in females than in males (Neugarten et al., 2000). Sex hormones may be responsible for this sex imbalance, as they directly or indirectly affect glomerular homeostasis. Indeed, female hormones such as estrogens have profound effects on the renin-angiotensin (Baylis, 2009, 2012; Xue et al., 2013; Herak-Kramberger et al., 2015; Pringle et al., 2015; Kafami et al., 2016; Lu et al., 2016; Mompeón et al., 2016) and endothelin (Kittikulsuth et al., 2013) systems, thus affecting glomerular hemodynamic. Other potential mechanisms include the receptor-mediated effects of estrogens on TGF-β signaling (Kim et al., 2014), which in turn regulates glomerular cell proliferation (Kim et al., 2016), matrix accumulation (Li et al., 2014), apoptosis (Doublier et al., 2011), and antioxidant capacity (Pérez-Torres et al., 2009; Matrai et al., 2016). In line with this evidence, selective estrogen receptor modulators ameliorated the progression of kidney disease in animal models (Gracelli et al., 2012; Tazumi et al., 2016) and postmenopausal females (Silbiger, 2009; Suzuki and Kondo, 2012; Pollow et al., 2015). Conversely, male hormones, such as testosterone, could make males more sensitive than females to similar or even lower immunological challenges (Iliescu and Reckelhoff, 2008). *Ad hoc* studies are needed to further investigate this intriguing issue.

### Limitations and strengths

In this observational study, we could not analyze B- and T-cell subpopulations that might be involved in the mechanism of antibody expression and modulation of response to Rituximab ^54^. The proportion of females was relatively small, which reflects the well-known unbalanced gender distribution of MN (Cravedi et al., 2011b; Ruggenenti et al., 2015; NIH Guidelines on the Inclusion of Women and Minorities as Subjects in Clinical Research-Amended, October 2001). The analysis of disease immunological activity was restricted to the monitoring of anti-PLA_2_R antibody titer at baseline and serial evaluations after exposure to Rituximab. Further studies are needed to evaluate the potential role of autoantibodies against specific PLA_2_R epitopes and multi-domain antibody recognition or “spreading” (Hoxha et al., 2012; De Vriese et al., 2017; Reinhard et al., 2020; Deng et al., 2021; Seitz-Polski et al., 2016)*.*

To our knowledge, this is the largest and longest study evaluating predictors of outcome in Rituximab-treated patients with MN. This strength enabled analyses powerful enough to confirm and extend previous evidence of the predictive value of well-established risk factors, such as the severity of proteinuria, and provide fully novel information about the additional and integrated predictive value of gender and anti-PLA_2_R antibody titer. The study population was homogeneous and was selected based on standard indications of Rituximab therapy (Ruggenenti et al., 2015). Some patients received the standard four-dose regimen, others the B-cell driven protocol. However, this was not an issue because we previously reported similar outcomes with both treatments (Cravedi et al., 2007)**.** Some patients received Rituximab as first-line therapy and others as second-line therapy. Again, this very unlikely introduced a bias in data interpretation because Rituximab was previously reported to be equally effective in both cases (Cravedi et al., 2011c)**.** No other immunosuppressive medication was allowed for at least 6 months before treatment administration and throughout the whole study period. Moreover, all patients received the same standardized supportive therapy, and their outcome was carefully monitored with pre-defined serial clinical and laboratory evaluations (Ruggenenti et al., 2015). The study endpoints were defined on standard criteria and adjudicated by a nephrologist (MP) who was blinded to patient characteristics. All laboratory measurements were centralized. These strengths increased the study power by reducing the risk of bias and random data fluctuations. On the contrary, whether study findings can be generalized to patients exposed to immunosuppressive treatments other than Rituximab needs further investigation.

## Conclusion

Our present findings clearly indicate that sex is a critical determinant in the clinical manifestation and progression of MN. The finding that females are more resilient to renal injury despite the same therapy with Rituximab and even in the face of a similar immunological challenge may open new avenues of interest to identify the molecular pathways underlying this sex-related protective effect. This issue is of critical interest in the attempt to identify novel targets amenable to pharmacological intervention and design more effective therapeutic approaches to halt or slow disease progression in patients who are poorly responsive to the currently available therapies, such as males with higher anti-PLA_2_R levels.

## Data Availability

The original contributions presented in the study are included in the article/supplementary material. Further inquiries can be directed to the corresponding author.
